# Editorial: From Functional Genomics to Biotechnology in Ornamental Plants

**DOI:** 10.3389/fpls.2019.00463

**Published:** 2019-04-16

**Authors:** Swee-Suak Ko, Akira Kanno, Raquel Sánchez-Pérez, Hsin-Hung Yeh, Annette Hohe, Mariana Mondragón-Palomino

**Affiliations:** ^1^Academia Sinica, ABRC/BCST, Taipei, Taiwan; ^2^Graduate School of Life Sciences, Tohoku University, Aoba-Ku, Sendai, Japan; ^3^CEBAS-CSIC, Murcia, Spain; ^4^Academia Sinica, ABRC, Taipei, Taiwan; ^5^Faculty of Landscaping, Horticulture and Forestry, University of Applied Sciences Erfurt, Erfurt, Germany; ^6^Department of Cell Biology and Plant Biochemistry, University of Regensburg, Regensburg, Germany

**Keywords:** ornamental plant, functional genomics, flower development, flower scent, flower color, genetic transformation, RNA-seq, CAM

“*Among roses there are many differences, in the number of petals, in roughness, in beauty of color, and in sweetness of scent. Most have five petals, but some have twelve or twenty, and some a great many more than these; for there are some, they say, which are even called ‘hundred-petalled.”’*

(Theophrastus, ca. 350 B.C.)

Ancient civilizations around the world already selected, cultivated and exchanged plants because of their distinctive or unusual flowers. This interest motivated, for instance, the domestication of *Dahlia* species by the Aztecs (Treviño de Castro et al., 2007) as well as the breeding of peonies in Imperial China and tulips in the Ottoman Empire (Kingsbury, 2009). Ornamental plant breeding experienced significant advances during the seventeenth and eighteenth centuries after the mechanisms of plant reproduction and hybridization were understood and commercial routes exchanged plants and their products around the globe. Scientific advances and international floriculture trade also drive the most recent breakthrough in ornamental plant sciences: The complete sequencing of the genomes of *Rosa chinensis, Aquilegia coerulea, Petunia* as well as *Phalaenopsis* and several other orchids (Cai et al., 2015; Bombarely et al., 2016; Chao et al., 2018; Filiault et al., 2018; Raymond et al., 2018), facilitate both basic scientific research and motivate the development of a biotechnological tool-kit for functional genomics. In this Research Topic, we present a primer into the changing face of this area and its new applications to floriculture in the twenty-first century.

## Flower Morphology, Color and Scent

Most of the 13 Original Research Articles in this Research Topic test hypotheses on the role of specific genes in flower organ development and organization or in the synthesis of flower scent and perianth color. Because genetic transformation is not feasible in most ornamental species, the three studies dealing with flower development tap on a diverse repertoire of morphological mutants. This strategy is not only helpful for developing biotechnological approaches in plant breeding, but it also offers fascinating insights into plant developmental biology and phylogeny.

In their article, Mitoma and Kanno analyzed the developmental genetics of *Habenaria radiata* “Ryokusei” (Orchidaceae), a mutant bearing greenish flowers and greenish sepaloid organs in place of the reproductive organs. Comparison of the patterns of expression of class C and class E MADS-box genes between flowers of *H. radiata* wild-type and the mutant suggest the suppressed expression of *HrSEP-1* might cause the unusual flower morphology. Further analyses of the mutant revealed a retrotransposon insertion on the first exon of *HrSEP-1*, which possibly explains its phenotype and suggests an essential role of this gene in the development of petals and labellum. Similarly to the study of Mitoma and Kanno; Cheon et al. associated the sepal-like petal phenotype of old ornamental azalea cultivars (*Rhododendron*, Ericaceae), to the insertion of an LTR-retrotransposon in a class B MADS-box gene. The authors found out that this insertion abolished the wild-type mRNA sequences of the *AP3*/*DEF* homologs, thus conferring these evergreen azaleas their sepaloid phenotype.

African violet (*Saintpaulia ionantha*) is characterized by bilateral flower symmetry. Hsu et al. investigated the molecular developmental genetics of perianth symmetry in this species by comparing the patterns of expression of *SiCYC1A* and *SiCYC1B*, orthologs of the transcription factor *CYCLOIDEA* (*CYC*), in the wild-type and each of two peloric forms with dorsalized actinomorphy (DA) or ventralized actinomorphy (VA). The authors showed heterotopic expression shifts of *SiCYC1A*/*1B* correlated with DA, whereas reduced expression of *SiCYC1A*/*1B* is associated with VA. The authors suggest the expression shifts might be caused by the activity of upstream trans-acting factors or epigenetic regulatory mechanisms, similar to those acting on *CYC* in the peloric mutant of *Linaria vulgaris* (Cubas et al., 1999).

Scent is a key determinant of the commercial value of pot and cut ornamental flowers. In this Research Topic, two contributions investigate the genes encoding enzymes mediating the synthesis of terpenes, volatile organic compounds that contribute to floral fragrance and herbivore inhibition. Chuang et al. compared the promoter fragments of geranyl diphosphate synthase (*GDPS*), a key enzyme for monoterpene biosynthesis, in 12 *Phalaenopsis* species or hybrids, to identify the genetic regulatory mechanisms behind floral scent. The authors concluded that in scented orchids the integrity of a dual repeat motif in the *GDPS* promoter is crucial for its activity. These findings are essential for the development of molecular markers needed to accelerate breeding of scented orchids and to study the genetic basis of scent-mediated orchid-pollinator co-evolution. In lily (*Lilium* “Siberia”), Zhang et al. investigated the contribution of rate-delimiting enzymes 1-deoxy-D-xylulose-5-phosphate synthase (LiDXS) and 1-deoxy-D-xylulose-5-phosphate reductoisomerase (LiDXR), to the synthesis of terpenes via the MEP pathway. The authors demonstrated via transgenic overexpression of these enzymes in tobacco flowers that they participate in the synthesis of mono- and diterpenes.

Besides their role in the composition of flower scent, naturally occurring terpenes and their derivatives have important pharmaceutical applications. Specifically, terpenoid indole alkaloids (TIAs), like vindoline are substrates for synthesis of drugs used in chemotherapy. Employing an approach based on RNA-seq, Liu et al. identified a novel AP2/ERF transcription factor gene (*CR1*) from *Catharanthus roseus* (rose periwinkle, Apocynaceae), which is involved in the regulation of TIAs synthesis. Functional gene analysis of *CR1* via virus-induced gene silencing (VIGS) demonstrated its downregulation increases the accumulation of vindoline and serpentine. The authors propose this finding could be applied to increase TIAs production.

Ornamentals with unusual flower colors have been selected and bred for centuries. However, the genetics of each species limits the palette of possible shades that can be obtained by hybridization and mutagenesis. The transfer of genes of interest between different species through genetic engineering (GE), already enabled the production of transgenic blue roses and carnations (Tanaka et al., 2010). Before any GE ornamental variety is commercialized, it must meet regulatory requirements involving molecular characterization and risk assessment (Chandler and Brugliera, 2011). In this number, Haselmair-Gosch et al. present detailed genetic, transcriptomic and biochemical analyses of orange flowering GE Petunias carrying a maize dihydroflavonol 4-reductase gene (*A1*). These cultivars had to be recalled after it was discovered they originated from transgenic parental lines. The study points out at one possible source of the transgenic construct as well as the fact that the *A1* gene involved encodes a lowly expressed enzyme with a low substrate acceptance. Thus, the unusual orange color results from the formation of pelargonidin in the context of a flavonol synthase and B-ring hydroxylation enzymes with strongly reduced activities.

## Epigenetic Factors Behind Plant Morphology

Understanding the contribution of epigenetic factors to plant phenotypic variance is essential to optimize molecular breeding and cultivation strategies and assess the impact of climate change in plant health and productivity. Ma et al. investigate the relationships between epigenetic and genetic variances, environmental factors and leaf dimensions of *Prunus mume* (ornamental prune, Rosaceae). Statistical analyses of genetic and epigenetic markers showed that epigenetic diversity was higher than genetic diversity and the increase or decrease of DNA methylation level might affect the expression of genes that determine leaf development and metabolism. The markers characterized facilitate considering epigenetic factors in molecular plant breeding.

## Regulation of Plant Physiology

*Phalaenopsis* was the first CAM species with a completely sequenced genome. In their contribution, Ping et al. investigated the genetic causes behind the different types of photosynthesis along the development of *Phalaenopsis aphrodite* (moth orchid). During tissue culture, the young seedling performs C3 photosynthesis and when leaves mature switches to CAM. The authors found that the gene encoding phosphoenolpyruvate carboxylase kinase (*PPCK*), is associated with the distinct performance of CAM photosynthesis during seedling ontogeny. This knowledge is relevant to optimize the micropropagation of this genus.

In this issue, two contributions focus on the molecular physiology of orchid seed germination. Mycorrhizal symbiosis is essential for the germination of orchid seeds, including those of *Dendrobium officinale*. Li et al. revealed the role of cell wall structural hydroxyproline-rich glycoproteins (HRGPs). The authors showed HRGPs are highly upregulated in symbiotically germinated protocorms of *D. officinale* and inhibition of their biosynthesis resulted in uncontrolled hyphae growth in protocorms, suggesting a relevant role in the regulation of symbiotic fungal colonization and compartmentalization.

Abscisic acid (ABA) has been shown to regulate stress responses, seed dormancy, and seed germination. Lee et al. characterized the expression of *PtNCED1* (9-cis-epoxycarotenoid dioxygenase), a candidate regulator of ABA biosynthesis, in orchid *Phaius tankervilliae*. This study revealed a relationship between *PtNCED1* expression and biosynthesis of ABA in developing orchid seeds, thus suggesting a link with the rate of germination and the content of ABA in seeds. This association is useful to further investigate the role of *PtNCED1* in seed dormancy.

## Approaches to Genetic Transformation

In this Research Topic, two studies describe techniques of genetic transformation which open up new possibilities for functional genomic analyses of *Cyclamen* (Primulaceae) and *Phalaenopsis in planta* or in cellular culture. Specifically, Ratjens et al. employed embryogenic callus cultures for *Agrobacterium*-mediated transformation of *Cyclamen persicum* and achieved transformation rates of up to 43%. Besides, the study generated fluorophore-based marker lines useful to follow the localization of auxin and reactive oxygen species concentrations during somatic embryogenesis. In order to gain an insight into genetic regulation in Orchidaceae, Lin et al. developed and tested a method of transient gene expression based on protoplast from *Phalaenopsis aphrodite* flowers. The feasibility of this approach was demonstrated by testing multiple hypotheses on gene regulation, protein-protein interactions, and subcellular localization.

## Author Contributions

MM-P provided the idea of the editorial and wrote the manuscript with contributions from S-SK, AK, RS-P, H-HY, and AH. All authors critically reviewed and approved the final manuscript.

### Conflict of Interest Statement

The authors declare that the research was conducted in the absence of any commercial or financial relationships that could be construed as a potential conflict of interest.

## References

[B1] Bombarely A. Moser M. Amrad A. Bao M. Bapaume L. Barry C. S.. (2016). Insight into the evolution of the Solanaceae from the parental genomes of *Petunia hybrida*. Nat. Plants 2, 16074. 10.1038/nplants.2016.74 27255838

[B2] Cai J. Liu X. Vanneste K. Proost S. Tsai W. C. Liu K. W. (2015). The genome sequence of the orchid *Phalaenopsis equestris*. Nat. Genet. 47, 65–72. 10.1038/ng.3149 25420146

[B3] Chandler S. F. Brugliera F. (2011). Genetic modification in floriculture. Biotechnol. Lett. 33, 207–214. 10.1007/s10529-010-0424-4 20882313

[B4] Chao Y. T. Chen W. C. Chen C. Y. Ho H. Y. Yeh C. H. Kuo Y. T.. (2018). Chromosome-level assembly, genetic and physical mapping of *Phalaenopsis aphrodite* genome provides new insights into species adaptation and resources for orchid breeding. Plant Biotechnol. J. 16, 2027–2041. 10.1111/pbi.12936 29704444 PMC6230949

[B5] Cubas P. Vincent C. Coen E. (1999). An epigenetic mutation responsible for natural variation in floral symmetry. Nature 401, 157–161. 10.1038/43657 10490023

[B6] Filiault D. L. Ballerini E. S. Mandáková T. Aköz G. Derieg N. J. Schmutz J.. (2018). The *Aquilegia* genome provides insight into adaptive radiation and reveals an extraordinarily polymorphic chromosome with a unique history. Elife 7:e36426. 10.7554/eLife.36426 30325307 PMC6255393

[B7] Kingsbury N. (2009). Hybrid: The History An Science Of Plant Breeding. Chicago: The University of Chicago Press.

[B8] Raymond O. Gouzy J. Just J. Badouin H. Verdenaud M. Lemainque A.. (2018). The *Rosa* genome provides new insights into the domestication of modern roses. Nat. Genet. 50, 772–777. 10.1038/s41588-018-0110-3 29713014 PMC5984618

[B9] Tanaka Y. Brugliera F. Kalc G. Senior M. Dyson B. Nakamura N.. (2010). Flower color modification by engineering of the flavonoid biosynthetic pathway: practical perspectives. Biosci. Biotechnol., Biochem. 74, 1760–1769. 10.1271/bbb.100358 20834175

[B10] Theophrastus (ca. 350 B.C.). Enquiry into Plants. Available online at: www.loebclassics.com/view/theophrastus-enquiry_plants/1916/pb_LCL079.39.xml.

[B11] Treviño de Castro G. Mera Ovando L. M. Bye Boettler R. Mejía Muñoz J. M. Laguna Cerda A. (2007). Historia de la dalia (Acocoxóchitl). La flor nacional de México. Chapingo: Publicación de difusión 1. SNICS-SAGARPA.

